# The Perfect Host: A Mouse Host Embryo Facilitating More Efficient Germ Line Transmission of Genetically Modified Embryonic Stem Cells

**DOI:** 10.1371/journal.pone.0067826

**Published:** 2013-07-02

**Authors:** Robert A. Taft, Benjamin E. Low, Shannon L. Byers, Stephen A. Murray, Peter Kutny, Michael V. Wiles

**Affiliations:** Technology Evaluation and Development, The Jackson Laboratory, Bar Harbor, Maine, United States of America; University of Melbourne, Australia

## Abstract

There is a continual need to improve efficiency in creating precise genetic modifications in mice using embryonic stem cells (ESCs). We describe a novel approach resulting in 100% germline transmission from competent injected ESCs. We developed an F1 mouse host embryo (Perfect Host, PH) that selectively ablates its own germ cells via tissue-specific induction of diphtheria toxin. This approach allows competent microinjected ESCs to fully dominate the germline, eliminating competition for this critical niche in the developing and adult animal. This is in contrast to conventional methods, where competition from host germ cells results in offspring derived from host cells and ESCs, necessitating extensive breeding of chimeras and genotyping to identify germline. The germline transmission process is also complicated by variability in the actual number of ESCs that colonize the germline niche and the proportion that are germline competent. To validate the PH approach we used ESC lines derived from 129 F1, BALB/cByJ, and BTBR backgrounds as well as an iPS line. Resulting chimeric males produced 194 offspring, all paternally derived from the introduced stem cells, with no offspring being derived from the host genome. We further tested this approach using eleven genetically modified C57BL/6N ESC lines (International Knockout Mouse Consortium). ESC germline transmission was observed in 9/11 (82%) lines using PH blastocysts, compared to 6/11 (55%) when conventional host blastocysts were used. Furthermore, less than 35% (83/240) of mice born in the first litters from conventional chimeras were confirmed to be of ESC-origin. By comparison, 100% (137/137) of the first litter offspring of PH chimeras were confirmed as ESC-derived. Together, these data demonstrate that the PH approach increases the probability of germline transmission and speeds the generation of ESC derived animals from chimeras. Collectively, this approach reduces the time and costs inherent in the production of genetically modified animals.

## Introduction

We are the product of our genes and their interaction with the environment. Genetic engineering of the mouse enables creation of models to aid our understanding these complex interactions, providing a deeper insight of biology and genetics through the systematic modification of the mouse genome and careful characterization of the resulting animals. The time and cost involved in generating genetically modified animals is a rate-limiting step in this process, creating a need to improve the efficiency with which genetically modified animals can be produced. Mouse embryonic stem cells (ESC) are currently the primary tool for precise modification of the mouse genome. Recent advances in ESC line culture has improved our ability to scale the production of modified cells; see review [1], and as exemplified by The Knockout Mouse Project (KOMP) [2]. However, it is the next stage, enhancing germline transmission of properly targeted ESC, which has lagged in development. When modified ESC are introduced into host embryos, they integrate into the host’s inner cell mass, thereby contributing to the three primary germ layers: endoderm, mesoderm, and ectoderm. They can also contribute to the primordial germ cell pool, producing germline transmitting chimeric animals [3], [4], [5], [6]. It is this successful generation of functional gametes from introduced ESC in sufficient quantity, i.e. germline transmission, which defines success and requires improvement.

The germ cell lineage becomes a distinguishable group of ∼8 migratory alkaline phosphatase positive cells by E7.0–E7.25 dpc. These cells develop to become primordial germ cells (PGC), then germ cells and ultimately gametes [7], [8], [9]. By E8.5, this PGC lineage-restricted population has expanded to ∼100 cells, increasing to ∼3000 germ cells by E11.5 as they reach the genital ridge [10]. In order for ESC lines introduced into host blastocysts to give rise to PGCs and eventually germline transmission, a number of poorly understood conditions and characteristics (i.e. competence) need to be fulfilled. These are thought to include: (i) ESCs being in the right place at the right time, integrating into the inner cell mass (ICM) developmental process, (ii) ESCs having an inherent (genetic and epigenetic) ability to become PGC and (iii) subsequently, develop to functional gametes. During embryonic development in conventional chimeras, both host and introduced stem cells compete for the developmental niche to eventually become gametes. A further complication in this development is that most ESCs lines are a mixed population of cells with differing genetic characteristics which have arisen during cell culture [11], [12]. These factors combine in chimeras leading to an unpredictable, variable (0–100%) germline contribution from introduced ESC. Although chimera coat color is often used as an indicator of probability of germline transmission of ESC, there is no direct correlation between *functional* colonization of the germline and the skin [12]. Nonetheless, the standard protocol followed by most laboratories is to breed visibly high coat color chimeras and wait for germline transmission to occur, or not. Unfortunately, it is not unusual for chimeras to be subfertile with respect for the desired genotype, with at times no ESC-derived animals being produced even after months of breeding. This “wait and see” approach detrimentally impacts project time, potentially delaying work which could have been repeated had there been an earlier and more definitive indicator of ESC germline transmission capability.

We approached these issues by reasoning that the host embryo and later, the adult mouse is simply a vehicle to generate gametes from introduced stem cells. Thus host-derived germ cells and gametes are simply a competitive distraction. Based on this premise, we developed a host embryo, referred here to as the “Perfect Host” (PH), in which endogenous germ cells are ablated during development thereby producing a sterile animal. These PH-derived mice are the sterile F1 offspring of two fertile parents. The germ cells of these F1 offspring are ablated by expression of tissue-specific Cre recombinase (inherited from one parent) driving a genomic excision that activates diphtheria toxin A (inherited from the other parent), occurring at ∼E10.5 [13], [14], [15]. This elimination of host germ cells avoids competition and allows exogenously added stem cells (i.e. microinjected into blastocysts) to exclusively dominate the germline early in development.

Since differences in the genetic background between ESCs and host embryos could influence competition and the ability of injected ESCs to colonize the germline, we verified the generality of this approach by injecting ESCs from four different genetic backgrounds into PH recipient blastocysts. With all four ESC lines, as well as with an iPS cell line, germline transmission occurred. Further, *all* offspring were derived paternally from the introduced stem cells, with no offspring being derived from the PH chimera paternal genome. The utility of this approach was further evaluated by comparing rates of transmission across eleven different genetically modified ESC clones injected into PH blastocysts versus conventional blastocysts. Using conventional host blastocysts only 6/11 of these ESC clones achieved germline transmission; in contrast, using PH blastocysts, 9/11 achieved germline transmission with all PH chimera offspring being derived from the microinjected ESCs.

Collectively, these data strongly suggest that using PH blastocysts as ESC recipients is a more efficient means of producing germline transmission than conventional hosts. Furthermore, by reducing competition with the host, the PH approach can potentially enhance germline recovery from poor, or low-level germline transmitting ESCs, providing overall logistic advantages in high-throughput genetically modified animal production.

## Methods

### Ethics Statement

The Institutional Animal Care and Use Committee of The Jackson Laboratory approved all procedures used in this study and all mice were maintained at The Jackson Laboratory (Bar Harbor, ME, USA) in strict accordance with all institutional protocols and the Guide for the Care and Use of Laboratory Animals.

### Mouse Strains for PH Blastocyst Production and Microinjection

Two strains of mice were used to produce PH F1 blastocysts: B6;129-*Gt(ROSA)26Sor^tm1(DTA)Mrc^*/J (JR010527), herein referred to as R26R^DTA^ [15]; and FVB-Tg(Ddx4-cre)1Dcas/J (JR006954), herein referred to as Vasa-Cre [16]. Before use, the Vasa-Cre strain was backcrossed onto C57BL6/J (JR000664). Both strains were maintained with their transgenes in a homozygous state so that all F1 offspring would inherit the genes to undergo Cre directed stop codon excision and diphtheria toxin A expression in germ cells. To obtain early embryos, female R26R^DTA^ mice were superovulated and mated with Vasa-Cre males. Reproductive tracts were collected and flushed at E2.5 dpc and harvested morula cultured overnight to blastocyst (E3.5 dpc). Conventional host blastocysts production used B6(Cg)-*Tyr^c-2J^*/J (JR000058) and embryos were obtained using the same methodology as above. For microinjection, 50–60 blastocysts per ESC clone were injected with 10–15 ESCs, depending upon embryo size. Approximately 11 blastocysts were implanted per pseudopregnant recipient CByB6F1/J female under isofluorane anesthesia, Carprofen was administered for post-surgical pain management. The same conditions were used for both conventional and PH blastocysts. Typically 50% of transferred blastocysts survived to term. At 7 weeks of age, all PH (putative chimeras) males were paired with C57BL/6NJ (JR005304) females. To determine the paternal genetic contribution of offspring derived from PH chimeras approximately 1 mm of tail or an ear punch was processed to crude DNA for genotyping. SNP genotyping was performed with a panel of 35 SNPs capable of unambiguously positively identifying the parental (germline) background of the ESC versus the host embryo [17]. This included five SNPs which can distinguish between C57BL/6J (PH) and C57BL/6N ESC lines. IVF was performed with either freshly isolated sperm or sperm previously isolated from vasa deferentia and cryopreserved [18], [19].

### Histology

For sections, tissues were collected in Bouin’s Fixative, dehydrated in graded alcohols and then xylene. Tissues were paraffin-embedded, sectioned, deparaffinized, and stained with PAS (Periodic Acid Schiff) and photographed.

### ESC Culture

The BtBr T^+^ Itpr3 ^tf^/J (JR002282) and BALB/cJ (JR000651)-derived ESC lines PB60.6 and PB150.18 ESC cells were isolated and provided by Predictive Biology, Inc. and are available through The Jackson Laboratory. The ESC line R1was derived from a 129X1/SvJ×129S1 cross [20]. The iPS cell line 9.48B (also known as 4.48B) was provided by the laboratory of Prof. Rudolf Jaenisch, and was derived from a cross of M26-M2rtTA×129sv; for complete lineage see [21]. Genetically modified IKMC C57BL/6N ESC lines were obtained from Eucomm, CSD and through Regeneron Pharmaceuticals Inc. VelociGene® program. ESC culture was conducted according to protocols provided by the ESC suppliers and as outlined in [22]. Briefly, CSD and EUCOMM ESC clones were grown in 10% FCS plus chemical inhibitors. Regeneron Pharmaceuticals supplied ESC were grown in VGB media. For complete culture protocols of KOMP clones see https://www.komp.org/protocols.php.

## Results

### Initial Characterization of PH Adult Animals

In seeking to develop a healthy host mouse which would completely ablate its germ cells early in development we test crossed a number of different tissue-specific cre promoter driver mouse strains with different loxP-based cell ablation approaches. The most effective strategy employed a strain carrying a Vasa (also know as DDX4 or Mvh) promoter driving cre recombinase (FVB-Tg(Ddx4-cre)1Dcas/J; abbreviated here to Vasa-Cre) crossed to a strain with a *floxed* STOP cassette adjacent to an attenuated diphtheria toxin (DTA) (B6;129-*Gt(ROSA)26Sor^tm1(DTA)Mrc^*/J; abbreviated here to R26R^DTA^). Previous work had demonstrated that the Vasa-Cre strain provided germ cell specific expression of Cre [16], [23]. Hence it was predicted that this cross would lead to offspring where cre-mediated recombination excision of the STOP occurs and DTA is expressed in the germ-cell lineage, resulting in cell ablation.

**Figure 1:**
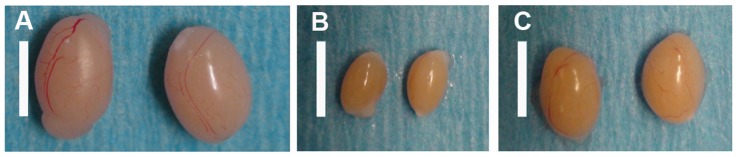
Dissected Testis. Testis were dissected from 8–12 week old sexually mature males; A) normal wild type C57Bl/6J mice, B) PH testis, where germ cells ablated, C) PH testis colonized (partially) by 129 F1 ESC line R1 derived germ cells. Scale bar equals 10 mm.

**Figure 2:**
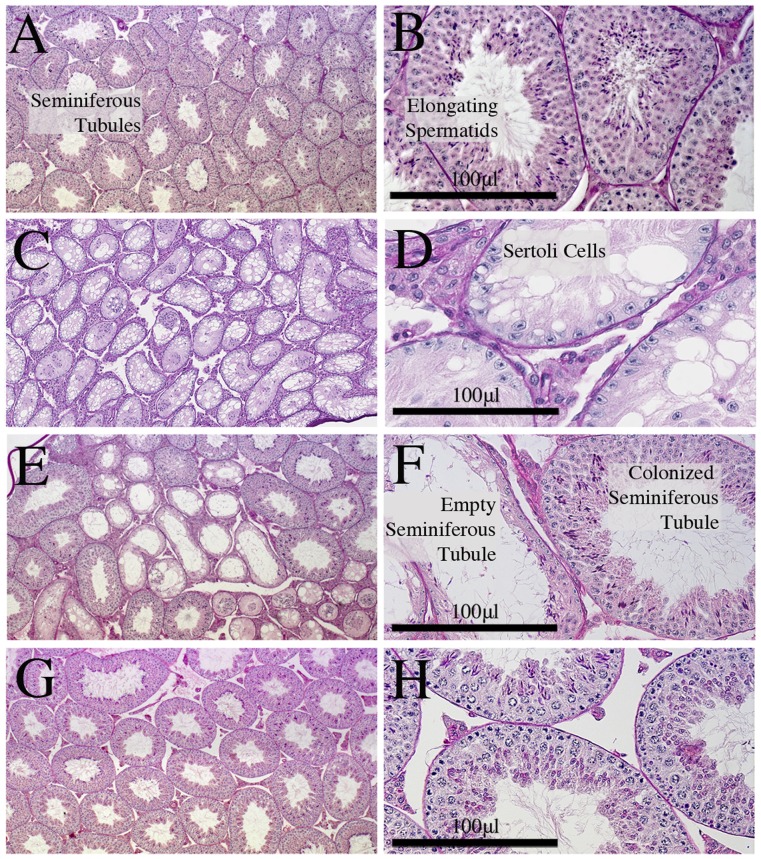
Sections of testis from wild type and F1 animals. Sections of testis at 5× and 20×, scale bar 100 micrometer: **A+B**) wild type C57Bl/6J testis, shows normal colonization of the testis seminiferous tubules with characteristic spermatogonia, spermatocytes, round spermatids and elongating spermatids; **C+D**) PH male, non colonized testis, these animals were sterile having no sperm in the vasa deferentia or epididymis, the seminiferous tubules are almost exclusively filled with Sertoli cells and are apparently devoid of sperm and earlier germ cell progenitors; **E+F**) PH male, partially colonized with differentiated derivatives of Balb/cJ derived ESC line PB150.18, shows partial colonization of the seminiferous tubules, this animal was fertile however, this phenotype was at times associated with reduced fertility (data not shown); **G+H**) PH male, well colonized testis with differentiated derivatives of the BtBr derived ESC line PB60.6, showing almost normal colonization of the testes.

When R26R^DTA^ females were crossed to Vasa-Cre males, F1 offspring were produced at the expected rate and appeared to be grossly normal. Coat color was agouti or less frequently black. As PH males grew towards sexual maturity it became obvious that their testes were of reduced size (∼12% volume of wild type), suggesting an absence of germ cell colonization; see Figure 1 panels A and B. Upon examination of mature F1 males vasa deferentia and epididymides, no sperm were observed (n = 5). Histological examination of testis confirmed the absence of sperm production and of detectable spermatogonial stem cells (SSC); see Figure 2 panels A and B. As expected, these males did not produce any offspring when mated (n = 5). These data demonstrate that this combination of strains leads to F1 males devoid of competing germ cells.

F1 PH females produced by this same cross displayed nearly complete infertility, with only vestigial ovaries and associated fat pad remaining (data not shown). However, during the course of these experiments we observed 2 of 99 PH mated females that did produce three litters of three to five offspring. These proved by SNP genotyping to be maternal host gamete derived. These data suggest that there are rare sporadic failures of cre-driven STOP excision in female PH mice which can lead to low level of host germ cell colonization and occasional “leakage”. No such failures have been observed in males (>200 PH males mated) and all further studies used only male PH animals.

Attempts to use the reciprocal cross, i.e. Vasa-Cre females×R26R^DTA^ males resulted in no offspring. Previous studies suggested that Cre protein is present in the oocyte of Vasa-Cre females and this would mediate a recombination event shortly after fertilization resulting in lethal expression of DTA [16].

### Colonization of the Germline in PH by ESC from Multiple Genetic Backgrounds

To validate the capabilities of the PH approach, we microinjected three different ESC lines and an iPS cell line derived from various genetic backgrounds into PH blastocysts. These data are summarized in Table 1.

**Table 1:** ESC used with PH approach.

Stem cell genetic background	ESC microinjected	Number of offspring
F1 129X1/SvJ×129S1	R1	*20
BtBr T+ Itpr3 tf/J	PB60.6	59
BALB/cJ	PB150.18	22
iPS line derived from C57BL/6×129sv crosses	9.48B	32

ESC lines and one iPS cell line from different genetic backgrounds were microinjected into E3.5 PH blastocysts, implanted into pseudopregnant females and brought to term. One to three of the resulting male PH chimeras were mated. All offspring were confirmed by SNPs to be paternally derived from the microinjected stem cell lines only.

* In the case of the R1 ESC line, sperm from a chimera was used directly in an IVF, yielding a further 75 offspring as a single cohort.

When ESCs of the 129 F1 line R1 were injected into PH blastocysts, two obvious coat color male chimeras were produced and paired with females. One animal proved fertile, siring 20 offspring, all of which were shown by SNP genotyping to be paternally derived from R1 ES cells. To test the hypothesis that PH chimeras could be used as a source of sperm exclusively derived from the introduced ESC and to rapidly expand a colony derived from microinjected ESC lines, this male was euthanized and sperm used in an IVF. The IVF was scaled to produce 75 offspring as a single cohort, see [19]. All 75 offspring were SNP genotyped and all were shown unequivocally to be paternally derived from the introduced ESC line R1. ESC PH blastocysts microinjections were repeated using a BtBr T^+^ Itpr3 ^tf^/J derived ESC line, PB60.6 and a BALB/cJ derived ESC cell line, PB150.18. Fertile PH male chimeras were produced from both lines and upon mating, generated 59 and 22 offspring respectively. As before, all PH chimera-derived offspring were shown by SNP genotyping to be paternally derived from the introduced stem cells, with no contribution detected from the PH genome.

Lastly, to test the developmental potential of iPS cells with the PH approach we microinjected the iPS cell line 9.48B. Eleven males were produced and all were used for mating. One of these males was fertile, giving rise to 32 pups. SNP genotyping confirmed that all offspring were derived from the introduced iPS cell line.

The testes of sexually mature PH male putative chimeras were sectioned and examined histologically for the presence of immature sperm and earlier progenitors. The males exhibited a continuum of seminiferous tubule germ cell colonization ranging from apparent full colonization, to varying levels of partial colonization with some empty tubules, to no visibly detectable colonization and empty tubules (Figure 2). Often, even though chimeras had smaller than normal testes suggestive of partial colonization, these animals appeared to be fertile in conventional mating (data not shown).

### Conventional Host vs PH, Comparative Germline Transmission of Genetically Modified ESCs

To determine if the PH approach improved the rate and efficiency of germline transmission from genetically modified ESCs over that of conventional hosts, we conducted comparative microinjection tests. Eleven different C57BL/6N-derived genetically modified ESC lines were obtained from the International Knockout Mouse Consortium (IKMC) (see Table 2). For the evaluation of germline transmission from chimeras using conventional host embryos (albino, B6(Cg)-*Tyr^c-2J^*/J), three males with the highest degree of coat color chimerism were used for breeding. As the Vasa-Cre×R26R^DTA^ cross produces agouti or black coat color offspring, all PH males were paired with C57BL/6NJ females for 6∼16 weeks.

**Table 2:** Summary of germline transmission with IKMC lines.

Gene	IKMC ESC Clone ID	ESC clone	Provider	Conventional Host Blastocysts ESC recipents		Perfect Host Blastocysts ESC recipents		
				GLT	offspring	GLT	offspring	$Fertile
Plk1	HEPD0663_7_E04	JM8A3.N1	Eucomm	*Failed*	0/215	*Failed*	0	0/12
Plk1	HEPD0663_7_G03	JM8A3.N1	Eucomm	*Failed*	0/96	*Failed*	0	0/13
Kdm6b	EPD0330_7_F03	JM8A1.N3	CSD	*Failed*	Sterile chimeras	*GLT	4/4	1/18
Sdha	EPD0670_1_C11	JM8A3.N1	CSD	*Failed*	0/43	*GLT	6/6	1/18
DRD2	HEPD0654_5_E11	JM8A3.N1	Eucomm	*Failed*	0/175	#GLT	11/11	1/13
Setd6	DEPD00513_4_C01	JM8A3.N1	CSD	GLT	31/70	GLT	44/44	3/17
Mrps25	12105B-B6	VGB6	Velocigene	GLT	4/15	GLT	91/91	7/25
Dnajc5g	15380A-C6	VGB6	Velocigene	GLT	4/22	GLT	18/18	1/12
Htr3b	10050A-F4	VGB6	Velocigene	GLT	13/42	GLT	77/77	7/14
Col18a1	15565A-F8	VGB6	Velocigene	GLT	9/27	GLT	25/25	2/12
Ghrhr	10030C-F5	VGB6	Velocigene	GLT	20/20	GLT	36/36	5/21
				**55%**	**81/725 (11%)**	**82%**	**308/308 (100%)**	**16%**

Summary of data obtained from comparison of conventional vs. PH microinjected with IKMC ESC clones. GLT (germline transmission) is defined as offspring being paternally derived from the introduced ESC as determined by coat color for conventional host derived animals or by SNP genotyping for PH derived animals. Approximately 50% of germline transmission offspring contained the modified allele (data not shown). With this set of 11 ESC clones germline transmission was obtained with 6/11 using conventional host blastocysts vs 9/11 using PH blastocysts as ESC recipients. If we consider only the first litters then using conventional host, 35% (83/240) of offspring in the first litters were donor ESC germline. In comparison, 100% (137/137) of PH males offspring in the first litters were donor germline.

$ Fertile refers to the number of PH putative chimeras which proved to be fertile.

* Two ESC lines produced PH chimeric males which were subfertile, producing no offspring by natural mating, however they were found to have sperm in the epididymis which were used post cryopreservation for IVF, providing successful germline transmission.

# The HEPD0654_5_E11 ESC line microinjected into PH gave germline transmission however, many of the pups, both carrying the modified or wild type allele were born dead or died shortly afterwards. However, survivors carrying the modified allele were obtained after a few litters.

By standard mating, both approaches gave similar germline transmission with 6/11 for conventional hosts and 7/11 for PH. However, if we consider efficiency and look at the rate of transmission in first litters then conventional host provided only 35% (83/240) ESC-derived offspring. In comparison, all first litters from PH males yielded 100% (137/137) ESC-derived offspring. Further, when putative male PH chimeras from the remaining four “failed” ESC lines were euthanized and the epididymides examined for sperm, sperm was found and isolated from two of the four ESC lines. This sperm was cryopreserved and used subsequently in an IVF, yielding germline transmission of both of these lines. As before, all PH derived offspring were confirmed to be paternally derived from the introduced ESC lines by SNP genotyping. These comparative data are summarized in Table 2 and reveal a greater rate of germline transmission from injected ESC when using PH blastocysts with 9 of 11 ESC lines transmitting, versus conventional blastocysts with only 6 of 11 ESC lines transmitting. Further, it was clearly apparent that the remaining two ESC lines had failed to contribute to the germline with the complete absence of sperm.

## Discussion

We describe the use of a Cre recombinase under the control of a Vasa promoter to generate embryos in which germ cells are ablated through germ cell specific expression of DTA, whilst maintaining an environment conducive to germ cell and gamete development. Chimeric animals born following the injection of ESC into these embryos are indistinguishable from chimeras created using conventional host embryos. However, unlike conventionally produced chimeras, offspring produced from PH chimeras are derived only from sperm originating from introduced ESC. This improves germline transmission rate and increases efficiency in generating animals from genetically modified ESCs.

Sexually mature PH females have residual ovaries, consistent with the view that ovarian development is driven by the presence of germ cells, without which the ovaries degenerate leaving only stromal tissue [8]. However, 2% of mated PH females produced a few PH derived offspring, suggesting that the Vasa promoter-driven cre excision event can occasionally fail during female germ cell and gamete development. To ascertain the actual fecundity of these females would require a more extensive study as very small litters are rarely brought to term by the dam.

PH adult males were found to be aspermic, with no visible sperm in the vasa deferentia or epididymides. Sectioning of the testis revealed histologically normal seminiferous tubule development, but no detectable sperm progenitors. The development of “empty” seminiferous tubules has been observed with the dominant white spotting (W/W) *c-Kit* mutation, where germ cells fail to colonize the testis [24]. These data are consistent with the view that germ cells in PH males are ablated early in development.

We tested if PH blastocysts could provide an environment where microinjected ESC could develop to PGCs that exclusively colonize the genital ridge. Conventional mating combined with IVF of PH chimeric males produced nearly 200 offspring all of which were paternally derived from the introduced stem cells, with no offspring derived from the PH recipient detected. Our data demonstrate: i) the PH strategy is effective for a number of distinct ESC genetic backgrounds, and ii) that production of host-derived offspring by PH males is absent and/or not significant. Further and importantly for production considerations, we also show that sperm of PH chimeras can be cryopreserved and used subsequently in IVF. These results validate the PH strategy as an effective mouse management tool, facilitating the rapid expansion of ESC-derived germline without interference from competing host-derived gametes.

Testes of fertile PH sexually mature males often showed a continuum of ESC derived SSC colonization of the seminiferous tubules, ranging from apparent full colonization, partial colonization, to apparently empty seminiferous tubules (Figure 2). Although we did not fully explore the relationship between seminiferous tubules colonization, sperm count, sperm quality and fertility, it was apparent that in most cases even partial colonization of testes is sufficient to provide fertility. This is consistent with previous published work using busulfan SSC depleted mice and SSC recolonization. These studies also demonstrated a threshold effect in SSC colonization and resulting sperm counts with apparently ∼20% of normal sperm counts representing a threshold value conferring fertility [25]. This effect may also be linked to possible epididymal storage and accumulation of sperm over time, which may in part compensate for reduced sperm production. Below this threshold there may not be enough healthy/viable sperm accumulated to confer fertility, however sperm isolation followed by IVF may overcome this.

We also evaluated the effectiveness of PH versus conventional blastocysts in creation of new mouse models by comparing germline transmission of eleven IKMC C57BL/6N-derived ESC clones, with both conventional and PH blastocysts as microinjection hosts. The PH-derived chimeras successfully transmitted 9/11 of the ESC lines tested, while conventional hosts transmitted only 6/11. Further, when the best conventional host chimeras were bred, less than 35% of offspring in the first litters were derived from the ESC germline. In comparison, 100% of the offspring from the first litters of the PH-derived chimeras were confirmed to be ESC-derived. With both approaches ∼50% of germline transmitting animals carried the modified alleles. Together, these data reveal that the PH approach uses less total resources while providing higher efficiency and probability of germline transmission of ESC lines.

Germline transmission of two of the IKMC ESC lines was obtained by using PH male chimeras as sperm donors for IVF. We believe that their fertility failure by natural mating was due to reduced sperm count due to poor colonization of the testis. The ability to isolate sperm from PH chimeras and cryopreserve it or use it directly in IVF provides further operational options. These include rescue of low level transmitting ESC (low sperm counts), as well as rapid offspring expansion directly from chimeras, significantly reducing time normally spent on breeding. This approach can also provide better logistical control of downstream operations and secures a cryopreserved stock. Additionally, when PH chimeras are devoid of sperm, even with visible coat color chimerism, the approach provides rapid closure of lines where the ESC are incapable of, or simply have not contributed to the germline. This represents savings in animal space, breeding, number of mice required and overall time in project execution.

Direct comparison of the conventional host vs PH and their respective abilities for germline transmission is difficult. In our analysis, initially only three males from conventional host showing the highest level of coat color chimerism were chosen to breed (although if these failed, lower level chimeras were bred if available). For PH, the coat color of the F1 often precluded selection of chimeras by coat color (black on agouti or black), and therefore all F1 males obtained were paired with females for 4 to ∼12 weeks to test for germline transmission. It is possible that conventional host chimeras produced from injection of the other five ESC would have eventually proved germline; however this was not observed after >500 offspring. In contrast, the PH chimeras revealed germline transmission rapidly, allowing timely closure for those lines that did not.

Our data show conclusively with this set of genetically modified ESC lines that the PH approach is more efficient than conventional hosts. However, there is a general caution with any approach using ESC, which may have increased relevance here. When ESCs integrate into the ICM a series of complex regulative, competitive and probabilistic interactions occur, resulting in a very limited pool of cells having the developmental predisposition and opportunity to give rise to PCGs. Crucially, the actual numbers of inner cell mass cells which give rise to PCGs is not known, but it is inferred to be quite low. Additionally, we know that ESC lines are karyotypically a mixed population, with individual cells within a population having inherently different germline capabilities [12], [26]. An example of this can be seen in frequent observations by us and others that even a high percentage of cells in the population with grossly normal karyotype is no guarantee of ESC germline transmission. For example, we observed a case of reduced fertility using the HEPD0654_5_E11IVF (DRD2) ESC line. This line had an 80% normal karyotype when injected, but with conventional host blastocysts failed to give germline. With PH chimeras ESC offspring, both wild type and those with the desired mutation were born dead. However, over time two litters did yield live offspring carrying the desired mutation. Also ESC lines EPD0330_7_F03 (Kdm6b) and EPD0670_1_C11 (Sdha), gave PH chimeras which were subfertile. Successful germline transmission of both of these ESC lines required their use as sperm donors for IVF. These observations are suggestive of compromised ESC genomes in the germline. As almost all ESC lines will have developed mutations, it is of crucial importance that F1 offspring be backcrossed even at the expense of slowing throughput, in an attempt to dilute cell culture derived mutations. We suggest that the PH approach more clearly highlights this challenge. Despite this caveat, the PH strategy will raise throughput and efficiency in the production of genetically modified mice.

### Final Comment

The success of the PH approach suggests a number of strategies enabling higher throughput of genetically modified animals from ESCs. In one scheme male PH animals can be paired with females at 7 weeks of age. After a further 2–4 weeks the males are sacrificed, checked for the presence of sperm in the vasa deferentia and epididymis and if present cryopreserved. The occurrence of sperm is strongly indicative that germline transmission can be achieved. If the previously paired females fail to produce pups, an IVF can be performed using this frozen sperm and scaled to produce the required number of animals. A further crucial benefit of this approach is that when PH animals fail to have sperm rapid closure can be reached and an informed decision can be made in regards as to how to proceed.
